# 4-(1*H*-Pyrrolo­[2,3-*b*]pyridin-2-yl)pyridine

**DOI:** 10.1107/S1600536813008672

**Published:** 2013-04-10

**Authors:** Ping-Hsin Huang, Yuh-Sheng Wen, Jiun-Yi Shen

**Affiliations:** a Cardinal Tien College of Healthcare & Management, Taipei, Taiwan 231, Republic of China; b Institute of Chemistry, Academia Sinica, Nankang, Taipei, Taiwan, Republic of China; c Department of Chemistry, National Taiwan University, Taipei, Taiwan, Republic of China

## Abstract

The asymmetric unit of the title compound, C_12_H_9_N_3_, contains two independent mol­ecules in which the dihedral angle between the pyridine and aza­indole rings are 8.23 (6) and 9.89 (2)°. In the crystal, both types of mol­ecule are connected by pairs of N—H—N hydrogen bonds into inversion dimers.

## Related literature

For the production of luminescent organic compounds, see: Liu *et al.* (2000 ▶); Parcerisa *et al.* (2008 ▶). For related structures, see: Huang *et al.* (2012 ▶).
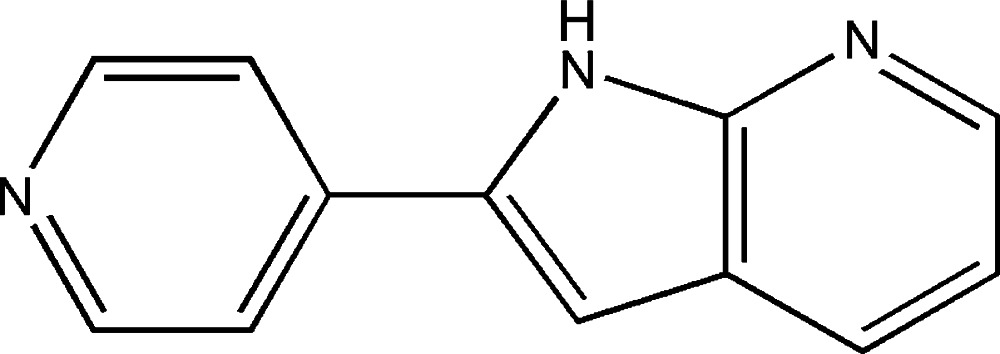


## Experimental

### 

#### Crystal data

C_12_H_9_N_3_
*M* *_r_* = 195.22
Triclinic, 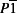

*a* = 6.5529 (5) Å
*b* = 10.0457 (8) Å
*c* = 14.5282 (11) Å
α = 83.372 (2)°
β = 86.697 (2)°
γ = 87.427 (2)°
*V* = 947.69 (13) Å^3^
*Z* = 4
Mo *K*α radiation
μ = 0.09 mm^−1^
*T* = 295 K
0.30 × 0.20 × 0.05 mm

#### Data collection

Bruker SMART APEX CCD area-detector diffractometer
Absorption correction: multi-scan (*SADABS*; Bruker, 2001 ▶) *T*_min_ = 0.975, *T*_max_ = 0.996
10193 measured reflections
3329 independent reflections
2573 reflections with *I* > 2σ(*I*)
*R*_int_ = 0.034

#### Refinement

*R*[*F* ^2^ > 2σ(*F* ^2^)] = 0.058
*wR*(*F* ^2^) = 0.124
*S* = 1.14
3329 reflections
271 parameters
H-atom parameters constrained
Δρ_max_ = 0.14 e Å^−3^
Δρ_min_ = −0.17 e Å^−3^

### 

Data collection: *SMART* (Bruker, 2001 ▶); cell refinement: *SAINT* (Bruker, 2001 ▶); data reduction: *SAINT*; program(s) used to solve structure: *SHELXS97* (Sheldrick, 2008 ▶); program(s) used to refine structure: *SHELXL97* (Sheldrick, 2008 ▶); molecular graphics: *ORTEP-3 for Windows* (Farrugia, 2012 ▶); software used to prepare material for publication: *WinGX* (Farrugia, 2012 ▶).

## Supplementary Material

Crystal structure: contains datablock(s) global, I. DOI: 10.1107/S1600536813008672/nc2308sup1.cif

Structure factors: contains datablock(s) I. DOI: 10.1107/S1600536813008672/nc2308Isup2.hkl

Supplementary material file. DOI: 10.1107/S1600536813008672/nc2308Isup3.cml

Additional supplementary materials:  crystallographic information; 3D view; checkCIF report

## Figures and Tables

**Table 1:** Hydrogen-bond geometry (Å, °)

*D*—H⋯*A*	*D*—H	H⋯*A*	*D*⋯*A*	*D*—H⋯*A*
N1—H1⋯N2^i^	0.86	2.22	3.061 (3)	167
N4—H4*A*⋯N5^ii^	0.86	2.22	3.066 (3)	169

Symmetry codes: (i) 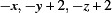; (ii) 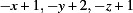.

**Fig. 1.**
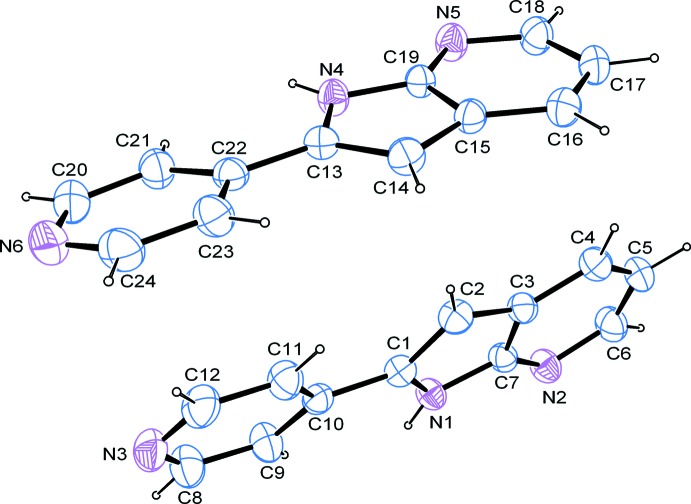
Molecular structure of the title compound with labeling and displacement ellipsoids drawn at the 30% probability level. H atoms are shown as small spheres of arbitrary radii.

## supplementary crystallographic information

### Comment

The title compound has been shown to be an precursor for the production of luminescent organic compound (Liu *et al.*, 2000). In the crystal structure of the title compound two crystallographically independent molecules are found which shows no large structural differences. Both molecules are nearly coplanar, the dihedral angles between the pyridine and the azaindole rings is 8.23 (6)° and 9.89 (2)° (Huang *et al.*, 2012). Each of these molecules is connected into centrosymmetrically dimers by intermolecular N—H—N hydrogen bonding.

### Experimental

The compound was synthesized by the following procedure (Parcerisa *et al.*, 2008). A solution of [3-(2-hydroxy-2-pyridin-4-yl-ethyl)-pyridin-2-yl]-carbamic acid *tert*-butyl ester (1 mmol and acetonitrile (12 ml) was cooled to ice temperature. Afterwards triethylamine (1.2 mmol) and trifluoromethanesulfonic anhydride (1.1 mmol) was added over a period of 5 min. The mixture was stirred at room temperature for 2 h, trifluoroacetic acid was added (1.5 mmol) and afterwards the mixture was heated under reflux for 1 h. The mixture was cooled to room temperature and neutralized using 2 N NaOH. The aqueous layer was extracted with ethyl ether and the organic extract was washed with brine and aqueous Na_2_SO_4_, dried and concentrated. The residue was purified by column chromatography using hexane/ethyl acetate (2:8) as eluent, followed by recrystallization in CH_2_Cl_2_ and hexane to give a white solid in 64% yield. Crystals suitable for X-ray diffraction were grown from a CH_2_Cl_2_ solution layered with hexane at room temperature. ^1^H NMR (CDCl_3_, 300 MHz): 8.62 (dd, 2 H, J= 1.0, 3.1 Hz), 8.29 (dd, 1 H, J= 1.0, 3.4 Hz), 8.00 (dd, 1 H, J = 1.0, 5.3 Hz), 7.72 (dd, 2 H, J = 1.0, 3.1 Hz), 7.13 (dd, 1 H, J= 3.4, 5.3 Hz), 6.99 (s, 1 H), Anal. Calcd for C_12_H_9_N_3_: C, 73.83; H, 4.65; N, 21.52. Found: C, 74.21; H, 4.40; N, 21.34.

### Refinement

H atoms were located in difference map but were positioned with idealized geometry and refined isotropic with *U*_iso_(H) = 1.2*U*_eq_(C,N).

### Crystal data

**Table:**

C_12_H_9_N_3_	*Z* = 4
*M**_r_* = 195.22	*F*(000) = 408
Triclinic, *P*1	*D*_x_ = 1.368 Mg m^−^^3^*D*_m_ = 1.368 Mg m^−^^3^*D*_m_ measured by not measured
Hall symbol: -P 1	Mo *K*α radiation, λ = 0.71073 Å
*a* = 6.5529 (5) Å	Cell parameters from 1585 reflections
*b* = 10.0457 (8) Å	θ = 2.6–23.3°
*c* = 14.5282 (11) Å	µ = 0.09 mm^−^^1^
α = 83.372 (2)°	*T* = 295 K
β = 86.697 (2)°	Plate, colorless
γ = 87.427 (2)°	0.30 × 0.20 × 0.05 mm
*V* = 947.69 (13) Å^3^	

### Data collection

**Table:**

Bruker SMART APEX CCD area-detector diffractometer	3329 independent reflections
Radiation source: fine-focus sealed tube	2573 reflections with *I* > 2σ(*I*)
Graphite monochromator	*R*_int_ = 0.034
ω scans	θ_max_ = 25.0°, θ_min_ = 1.4°
Absorption correction: multi-scan (*SADABS*; Bruker, 2001)	*h* = −7→7
*T*_min_ = 0.975, *T*_max_ = 0.996	*k* = −11→11
10193 measured reflections	*l* = −17→17

### Refinement

**Table:**

Refinement on *F*^2^	Primary atom site location: structure-invariant direct methods
Least-squares matrix: full	Secondary atom site location: difference Fourier map
*R*[*F*^2^ > 2σ(*F*^2^)] = 0.058	Hydrogen site location: inferred from neighbouring sites
*wR*(*F*^2^) = 0.124	H-atom parameters constrained
*S* = 1.14	*w* = 1/[σ^2^(*F*_o_^2^) + (0.0442*P*)^2^ + 0.126*P*] where *P* = (*F*_o_^2^ + 2*F*_c_^2^)/3
3329 reflections	(Δ/σ)_max_ < 0.001
271 parameters	Δρ_max_ = 0.14 e Å^−^^3^
0 restraints	Δρ_min_ = −0.17 e Å^−^^3^

### Special details

**Table:**

Geometry. All e.s.d.'s (except the e.s.d. in the dihedral angle between two l.s. planes) are estimated using the full covariance matrix. The cell e.s.d.'s are taken into account individually in the estimation of e.s.d.'s in distances, angles and torsion angles; correlations between e.s.d.'s in cell parameters are only used when they are defined by crystal symmetry. An approximate (isotropic) treatment of cell e.s.d.'s is used for estimating e.s.d.'s involving l.s. planes.
Refinement. Refinement of *F*^2^ against ALL reflections. The weighted *R*-factor *wR* and goodness of fit *S* are based on *F*^2^, conventional *R*-factors *R* are based on *F*, with *F* set to zero for negative *F*^2^. The threshold expression of *F*^2^ > σ(*F*^2^) is used only for calculating *R*-factors(gt) *etc*. and is not relevant to the choice of reflections for refinement. *R*-factors based on *F*^2^ are statistically about twice as large as those based on *F*, and *R*- factors based on ALL data will be even larger.

### Fractional atomic coordinates and isotropic or equivalent isotropic displacement parameters (Å^2^)

**Table:**

	*x*	*y*	*z*	*U*_iso_*/*U*_eq_	
N1	0.2449 (3)	0.99700 (17)	0.92427 (12)	0.0443 (5)	
H1	0.1787	1.0568	0.9532	0.053*	
N2	−0.0037 (3)	0.82637 (19)	0.94609 (13)	0.0523 (5)	
N3	0.7664 (4)	1.3698 (2)	0.86462 (16)	0.0704 (6)	
N4	0.7252 (3)	0.97632 (17)	0.57580 (12)	0.0452 (5)	
H4A	0.6651	1.0437	0.5450	0.054*	
N5	0.4869 (3)	0.80868 (19)	0.55706 (13)	0.0524 (5)	
N6	1.2240 (4)	1.3404 (2)	0.62691 (16)	0.0710 (6)	
C1	0.4379 (3)	1.0098 (2)	0.88094 (14)	0.0423 (5)	
C2	0.4911 (3)	0.8951 (2)	0.84219 (15)	0.0493 (6)	
H2	0.6131	0.8780	0.8089	0.059*	
C3	0.3285 (3)	0.8067 (2)	0.86152 (15)	0.0435 (5)	
C4	0.2881 (4)	0.6784 (2)	0.84216 (16)	0.0548 (6)	
H4	0.3826	0.6290	0.8083	0.066*	
C5	0.1035 (4)	0.6273 (2)	0.87473 (17)	0.0576 (7)	
H5	0.0713	0.5418	0.8630	0.069*	
C6	−0.0344 (4)	0.7025 (2)	0.92484 (17)	0.0589 (7)	
H6	−0.1580	0.6644	0.9454	0.071*	
C7	0.1773 (3)	0.8734 (2)	0.91330 (14)	0.0416 (5)	
C8	0.5755 (5)	1.3615 (3)	0.90108 (19)	0.0712 (8)	
H8	0.5140	1.4376	0.9232	0.085*	
C9	0.4636 (4)	1.2481 (2)	0.90816 (18)	0.0603 (7)	
H9	0.3301	1.2493	0.9337	0.072*	
C10	0.5497 (3)	1.1326 (2)	0.87731 (14)	0.0445 (5)	
C11	0.7495 (4)	1.1396 (2)	0.84044 (16)	0.0561 (6)	
H11	0.8162	1.0644	0.8193	0.067*	
C12	0.8479 (4)	1.2578 (3)	0.83527 (18)	0.0678 (7)	
H12	0.9811	1.2597	0.8094	0.081*	
C13	0.9063 (3)	0.9796 (2)	0.61960 (14)	0.0424 (5)	
C14	0.9524 (3)	0.8542 (2)	0.66219 (15)	0.0485 (6)	
H14	1.0656	0.8293	0.6969	0.058*	
C15	0.7970 (3)	0.7688 (2)	0.64396 (14)	0.0436 (5)	
C16	0.7536 (4)	0.6348 (2)	0.66723 (16)	0.0554 (6)	
H16	0.8395	0.5771	0.7035	0.066*	
C17	0.5792 (4)	0.5904 (2)	0.63473 (17)	0.0573 (6)	
H17	0.5454	0.5012	0.6486	0.069*	
C18	0.4535 (4)	0.6788 (2)	0.58133 (17)	0.0557 (6)	
H18	0.3366	0.6450	0.5607	0.067*	
C19	0.6574 (3)	0.8490 (2)	0.58945 (14)	0.0415 (5)	
C20	1.0412 (5)	1.3402 (3)	0.59264 (18)	0.0692 (8)	
H20	0.9818	1.4225	0.5701	0.083*	
C21	0.9327 (4)	1.2275 (2)	0.58795 (16)	0.0566 (6)	
H21	0.8044	1.2350	0.5631	0.068*	
C22	1.0152 (3)	1.1027 (2)	0.62031 (14)	0.0444 (5)	
C23	1.2075 (4)	1.1013 (2)	0.65608 (17)	0.0559 (6)	
H23	1.2712	1.0205	0.6787	0.067*	
C24	1.3034 (4)	1.2199 (3)	0.65792 (18)	0.0672 (7)	
H24	1.4319	1.2159	0.6824	0.081*	

### Atomic displacement parameters (Å^2^)

**Table:**

	*U*^11^	*U*^22^	*U*^33^	*U*^12^	*U*^13^	*U*^23^
N1	0.0419 (10)	0.0413 (11)	0.0508 (11)	−0.0013 (8)	0.0011 (9)	−0.0113 (8)
N2	0.0454 (11)	0.0507 (12)	0.0623 (13)	−0.0102 (9)	0.0003 (9)	−0.0112 (9)
N3	0.0765 (16)	0.0626 (16)	0.0714 (15)	−0.0247 (13)	−0.0063 (12)	0.0053 (12)
N4	0.0435 (11)	0.0392 (11)	0.0524 (11)	−0.0022 (8)	−0.0088 (9)	−0.0003 (8)
N5	0.0511 (12)	0.0480 (12)	0.0583 (12)	−0.0112 (9)	−0.0108 (9)	0.0003 (9)
N6	0.0781 (17)	0.0669 (16)	0.0707 (15)	−0.0277 (13)	0.0007 (13)	−0.0125 (12)
C1	0.0354 (12)	0.0475 (14)	0.0435 (13)	−0.0009 (10)	−0.0020 (10)	−0.0034 (10)
C2	0.0432 (13)	0.0539 (15)	0.0498 (14)	0.0005 (11)	0.0038 (11)	−0.0054 (11)
C3	0.0457 (13)	0.0409 (13)	0.0441 (13)	0.0020 (10)	−0.0037 (10)	−0.0057 (10)
C4	0.0627 (16)	0.0474 (15)	0.0542 (15)	0.0029 (12)	0.0004 (12)	−0.0089 (11)
C5	0.0703 (17)	0.0414 (14)	0.0629 (16)	−0.0094 (12)	−0.0066 (13)	−0.0096 (11)
C6	0.0570 (15)	0.0540 (16)	0.0674 (17)	−0.0163 (12)	−0.0014 (13)	−0.0095 (13)
C7	0.0408 (12)	0.0412 (13)	0.0439 (13)	−0.0038 (10)	−0.0064 (10)	−0.0062 (10)
C8	0.080 (2)	0.0510 (17)	0.083 (2)	−0.0045 (14)	−0.0019 (16)	−0.0076 (14)
C9	0.0568 (15)	0.0499 (16)	0.0747 (18)	−0.0087 (12)	0.0038 (13)	−0.0109 (13)
C10	0.0477 (13)	0.0461 (14)	0.0394 (12)	−0.0052 (10)	−0.0064 (10)	0.0002 (10)
C11	0.0500 (14)	0.0573 (16)	0.0607 (16)	−0.0104 (12)	0.0045 (12)	−0.0049 (12)
C12	0.0593 (17)	0.076 (2)	0.0665 (18)	−0.0216 (15)	0.0066 (13)	0.0018 (15)
C13	0.0394 (12)	0.0457 (14)	0.0423 (12)	−0.0011 (10)	−0.0029 (10)	−0.0059 (10)
C14	0.0448 (13)	0.0499 (15)	0.0509 (14)	0.0019 (11)	−0.0111 (11)	−0.0034 (11)
C15	0.0465 (13)	0.0395 (13)	0.0439 (13)	0.0006 (10)	−0.0027 (10)	−0.0016 (10)
C16	0.0632 (16)	0.0461 (15)	0.0559 (15)	0.0016 (12)	−0.0070 (12)	−0.0009 (11)
C17	0.0707 (17)	0.0407 (14)	0.0604 (16)	−0.0123 (12)	−0.0045 (13)	−0.0010 (11)
C18	0.0556 (15)	0.0524 (16)	0.0595 (15)	−0.0157 (12)	−0.0078 (12)	−0.0004 (12)
C19	0.0431 (12)	0.0385 (13)	0.0429 (12)	−0.0062 (10)	−0.0006 (10)	−0.0040 (9)
C20	0.087 (2)	0.0538 (17)	0.0668 (18)	−0.0156 (15)	−0.0081 (16)	0.0007 (13)
C21	0.0595 (15)	0.0505 (15)	0.0601 (16)	−0.0079 (12)	−0.0115 (12)	−0.0013 (12)
C22	0.0440 (13)	0.0497 (14)	0.0403 (13)	−0.0053 (10)	0.0007 (10)	−0.0083 (10)
C23	0.0494 (14)	0.0582 (16)	0.0618 (16)	−0.0072 (12)	−0.0079 (12)	−0.0093 (12)
C24	0.0570 (16)	0.082 (2)	0.0658 (18)	−0.0206 (15)	−0.0063 (13)	−0.0156 (15)

### Geometric parameters (Å, º)

**Table:**

N1—C7	1.366 (2)	C8—H8	0.9300
N1—C1	1.384 (2)	C9—C10	1.378 (3)
N1—H1	0.8600	C9—H9	0.9300
N2—C7	1.338 (3)	C10—C11	1.387 (3)
N2—C6	1.343 (3)	C11—C12	1.368 (3)
N3—C12	1.329 (3)	C11—H11	0.9300
N3—C8	1.332 (3)	C12—H12	0.9300
N4—C19	1.362 (3)	C13—C14	1.367 (3)
N4—C13	1.382 (2)	C13—C22	1.457 (3)
N4—H4A	0.8600	C14—C15	1.416 (3)
N5—C19	1.334 (3)	C14—H14	0.9300
N5—C18	1.336 (3)	C15—C16	1.388 (3)
N6—C20	1.324 (3)	C15—C19	1.408 (3)
N6—C24	1.336 (3)	C16—C17	1.373 (3)
C1—C2	1.362 (3)	C16—H16	0.9300
C1—C10	1.457 (3)	C17—C18	1.384 (3)
C2—C3	1.413 (3)	C17—H17	0.9300
C2—H2	0.9300	C18—H18	0.9300
C3—C4	1.390 (3)	C20—C21	1.374 (3)
C3—C7	1.403 (3)	C20—H20	0.9300
C4—C5	1.373 (3)	C21—C22	1.384 (3)
C4—H4	0.9300	C21—H21	0.9300
C5—C6	1.381 (3)	C22—C23	1.390 (3)
C5—H5	0.9300	C23—C24	1.375 (3)
C6—H6	0.9300	C23—H23	0.9300
C8—C9	1.373 (3)	C24—H24	0.9300
			
C7—N1—C1	108.49 (17)	C12—C11—H11	120.1
C7—N1—H1	125.8	C10—C11—H11	120.1
C1—N1—H1	125.8	N3—C12—C11	124.5 (3)
C7—N2—C6	113.4 (2)	N3—C12—H12	117.7
C12—N3—C8	115.3 (2)	C11—C12—H12	117.7
C19—N4—C13	108.82 (17)	C14—C13—N4	108.76 (19)
C19—N4—H4A	125.6	C14—C13—C22	128.8 (2)
C13—N4—H4A	125.6	N4—C13—C22	122.39 (19)
C19—N5—C18	113.62 (19)	C13—C14—C15	107.79 (19)
C20—N6—C24	115.3 (2)	C13—C14—H14	126.1
C2—C1—N1	108.75 (19)	C15—C14—H14	126.1
C2—C1—C10	129.1 (2)	C16—C15—C19	117.3 (2)
N1—C1—C10	122.06 (19)	C16—C15—C14	136.3 (2)
C1—C2—C3	108.01 (19)	C19—C15—C14	106.40 (18)
C1—C2—H2	126.0	C17—C16—C15	117.6 (2)
C3—C2—H2	126.0	C17—C16—H16	121.2
C4—C3—C7	117.2 (2)	C15—C16—H16	121.2
C4—C3—C2	136.3 (2)	C16—C17—C18	119.9 (2)
C7—C3—C2	106.46 (19)	C16—C17—H17	120.0
C5—C4—C3	117.6 (2)	C18—C17—H17	120.0
C5—C4—H4	121.2	N5—C18—C17	125.2 (2)
C3—C4—H4	121.2	N5—C18—H18	117.4
C4—C5—C6	120.1 (2)	C17—C18—H18	117.4
C4—C5—H5	119.9	N5—C19—N4	125.41 (19)
C6—C5—H5	119.9	N5—C19—C15	126.4 (2)
N2—C6—C5	125.0 (2)	N4—C19—C15	108.23 (18)
N2—C6—H6	117.5	N6—C20—C21	124.8 (3)
C5—C6—H6	117.5	N6—C20—H20	117.6
N2—C7—N1	125.10 (19)	C21—C20—H20	117.6
N2—C7—C3	126.6 (2)	C20—C21—C22	119.7 (2)
N1—C7—C3	108.29 (18)	C20—C21—H21	120.2
N3—C8—C9	124.4 (3)	C22—C21—H21	120.2
N3—C8—H8	117.8	C21—C22—C23	116.1 (2)
C9—C8—H8	117.8	C21—C22—C13	122.5 (2)
C8—C9—C10	119.7 (2)	C23—C22—C13	121.3 (2)
C8—C9—H9	120.1	C24—C23—C22	119.7 (2)
C10—C9—H9	120.1	C24—C23—H23	120.1
C9—C10—C11	116.3 (2)	C22—C23—H23	120.1
C9—C10—C1	122.5 (2)	N6—C24—C23	124.3 (2)
C11—C10—C1	121.1 (2)	N6—C24—H24	117.8
C12—C11—C10	119.7 (2)	C23—C24—H24	117.8
			
C7—N1—C1—C2	0.5 (2)	C19—N4—C13—C14	−0.7 (2)
C7—N1—C1—C10	177.80 (18)	C19—N4—C13—C22	−178.17 (19)
N1—C1—C2—C3	−0.2 (2)	N4—C13—C14—C15	0.6 (2)
C10—C1—C2—C3	−177.3 (2)	C22—C13—C14—C15	177.8 (2)
C1—C2—C3—C4	179.9 (2)	C13—C14—C15—C16	−179.2 (3)
C1—C2—C3—C7	−0.1 (2)	C13—C14—C15—C19	−0.3 (2)
C7—C3—C4—C5	0.3 (3)	C19—C15—C16—C17	0.5 (3)
C2—C3—C4—C5	−179.7 (2)	C14—C15—C16—C17	179.3 (2)
C3—C4—C5—C6	0.0 (4)	C15—C16—C17—C18	−0.4 (4)
C7—N2—C6—C5	0.4 (3)	C19—N5—C18—C17	0.0 (3)
C4—C5—C6—N2	−0.3 (4)	C16—C17—C18—N5	0.1 (4)
C6—N2—C7—N1	179.2 (2)	C18—N5—C19—N4	−179.1 (2)
C6—N2—C7—C3	−0.1 (3)	C18—N5—C19—C15	0.2 (3)
C1—N1—C7—N2	−179.9 (2)	C13—N4—C19—N5	180.0 (2)
C1—N1—C7—C3	−0.5 (2)	C13—N4—C19—C15	0.6 (2)
C4—C3—C7—N2	−0.2 (3)	C16—C15—C19—N5	−0.5 (3)
C2—C3—C7—N2	179.8 (2)	C14—C15—C19—N5	−179.6 (2)
C4—C3—C7—N1	−179.60 (18)	C16—C15—C19—N4	178.96 (19)
C2—C3—C7—N1	0.4 (2)	C14—C15—C19—N4	−0.2 (2)
C12—N3—C8—C9	−0.9 (4)	C24—N6—C20—C21	0.4 (4)
N3—C8—C9—C10	0.8 (4)	N6—C20—C21—C22	−0.3 (4)
C8—C9—C10—C11	0.2 (4)	C20—C21—C22—C23	−0.1 (3)
C8—C9—C10—C1	−178.6 (2)	C20—C21—C22—C13	178.4 (2)
C2—C1—C10—C9	170.0 (2)	C14—C13—C22—C21	−168.3 (2)
N1—C1—C10—C9	−6.7 (3)	N4—C13—C22—C21	8.6 (3)
C2—C1—C10—C11	−8.7 (4)	C14—C13—C22—C23	10.1 (3)
N1—C1—C10—C11	174.5 (2)	N4—C13—C22—C23	−173.0 (2)
C9—C10—C11—C12	−0.9 (3)	C21—C22—C23—C24	0.2 (3)
C1—C10—C11—C12	177.9 (2)	C13—C22—C23—C24	−178.2 (2)
C8—N3—C12—C11	0.0 (4)	C20—N6—C24—C23	−0.2 (4)
C10—C11—C12—N3	0.9 (4)	C22—C23—C24—N6	−0.1 (4)

### Hydrogen-bond geometry (Å, º)

**Table:**

*D*—H···*A*	*D*—H	H···*A*	*D*···*A*	*D*—H···*A*
N1—H1···N2^i^	0.86	2.22	3.061 (3)	167
N4—H4*A*···N5^ii^	0.86	2.22	3.066 (3)	169

Symmetry codes: (i) −*x*, −*y*+2, −*z*+2; (ii) −*x*+1, −*y*+2, −*z*+1.
